# A comparative study of the risk assessment and heavy metal contamination of coastal sediments in the Red sea, Egypt, between the cities of El-Quseir and Safaga

**DOI:** 10.1186/s12932-024-00086-8

**Published:** 2024-05-03

**Authors:** Ahmed R. Elgendy, Abd El Mohsen S. El Daba, Mohamed A. El-Sawy, Ahmed E. Alprol, Ghada Y. Zaghloul

**Affiliations:** 1 Geology Lab National Institute of Oceanography and Fisheries, Ashmoun, Egypt; 2 Marine Chemistry Lab National Institute of Oceanography and Fisheries, Hurghada, Egypt; 3 Marine Pollution Lab National Institute of Oceanography and Fisheries, Alexandria, Egypt

**Keywords:** Coastal sediment, Red Sea, El-Quseir and Safaga, Heavy metal pollution, Contamination factor, Potential cological risk index

## Abstract

This study aimed to assess the influence of pollution on the quality of sediments and the risks associated with El-Qusier and Safaga Cities, Red Sea, Egypt, during 2021, divided into four sectors, using multiple pollution indices. To achieve that, we evaluated the metal pollution index (MPI), contamination factor (Cf), pollution load index (PLI), contamination security index (CSI), and anthropogenicity (Anp%). Moreover, carcinogenic and non-carcinogenic risks are used for human health hazards. Results indicated that Mn and Fe recorded the highest concentrations, whereas Cd had the lowest. El-Quseir City sediments were found the following metal ions: Fe > Mn > Ni > Zn > Cu > Co > Pb > Cd, where the order in the Safaga City was: Fe > Mn > Zn > Ni > Cu > Pb > Co > Cd. MPI > 1, this is alarming in the study area due to heavy metal pollution. In addition, C_*f*_ < 1 in all metals except Cd with contamination degree CD ranged from low to considerable contamination in El-Qusier city. In contrast, contamination ranged from significant to very high in Safaga city. PLI < 1 is lower than the reference at all monitored stations. CSI values ranged from relatively low to moderate. Besides Cd, data reflect each element's low environmental danger (Eri^Me^40). This study's risk index (RI) is low to moderate in Sector 1 and high to extremely high in Sector 2. HQ and HI index < 1 means it is safe for human health in order: HI _ingestion_ > HI _dermal_. CSR for different pathways was recorded as dermal > ingestion, in which total CSR for all paths is considered harmful, and the cancer risk is troublesome and higher than the reference ranges of 1 × 10^–6^–1 × 10^–4^. In conclusion, the examined heavy metals provide environmental hazards across the assessed locations.

**Supplementary Information:**

The online version contains supplementary material available at 10.1186/s12932-024-00086-8.

## Introduction

Egypt’s Red Sea coast is over 1250 km long from Suez to the Sudanese border. The marine environment is crucial to human survival because of the food it produces and the ecosystem services it offers [41]. Safaga, an Egyptian municipality, is positioned on the southern coastline of the Red Sea, 53 km^2^ (33 miles) south of Hurghada. Safaga Port is a small port that stands out due to the presence of a tourism zone with numerous bungalows and recreational amenities*.* According to medical research conducted by Riegl and Piller [47] Safaga has gained recognition as a noteworthy location for therapeutic tourism, mainly owing to its ability to attract international tourists. El-Quseir is a prominent coastal municipality located in the eastern region of Egypt, playing a crucial role as a significant conduit connecting Egypt to the Red Sea. The area’s geographical coordinates are around 130 km north of Marsa Alam and 138 km south of Hurghada.

Heavy metals are considered harmful contaminants in aquatic environments due to their toxicity, persistence, and bioaccumulation issues [19]. Many biogeochemical processes and human causes influence the accumulation of heavy metals in sediments [43, 44]. Heavy metals can penetrate the marine ecosystem by natural weathering of rocks along the coast and estuaries, as well as human activities including tourism, mining, household garbage, ship traffic, car emissions, and open solid waste disposal [4, 8]. Furthermore, erosion of rocks in the hinterland highlands, particularly during floods, is a significant source of this problem [41].

Metals can move between water and sediments through ion exchange, metal substitution, adsorption, and dissolution. The distribution of possibly harmful metals in sediments along the Red Sea shoreline can reveal necessary information about the ecosystem’s environmental status [42]. Surface sediments in each site include varying quantities of trace metals, which can reflect the level of pollution, its sources and the ecosystem's consequences [59]. Heavy metals have both carcinogenic and non-carcinogenic hazards. Therefore, it is imperative to thoroughly analyze the potential health hazards associated with these factors [43, 46].

Risk assessment has recently become popular and widely used to determine what might happen if heavy metals enter and build up in sediments. People are interested in this subject because it looks at the ecological assessment of human health as a big, long-term project with clear goals for prevention, management, reduction, and long-term solutions [48], Mohamed et al., 2022). The introduction of heavy metals into the human body by skin absorption, inhalation, and oral ingestion continues to be a subject of concern within human health risk assessment [9]. In recent years, risk assessment has emerged as one of the most important and commonly used methodologies for assessing the possible consequences of the presence and accumulation of HM in sediments.

This study aims to find out how human activities affect the amount of heavy metals in the surface sediments of the Egyptian cities of El-Quseir and Safaga, which are on the Red Sea. Furthermore, it examines the possible ecological and human health risks linked to contaminated sediments. Various important specialized pollution indicators were utilized and scrutinized to accomplish these aims in assessing sediment quality. The indices encompassed in this study comprise the contamination factor (C_*f*_), the metal pollution index (MPI), the pollution load index (PLI), the contamination security index (CSI), anthropogenic (Anp%) and the individual and total risk index (Eri and RI). Human hazards are also based on carcinogenic (CSR) and non-carcinogenic (HQ and HI) impacts.

## Materials and methods

### Study area

**Fig. 1:**
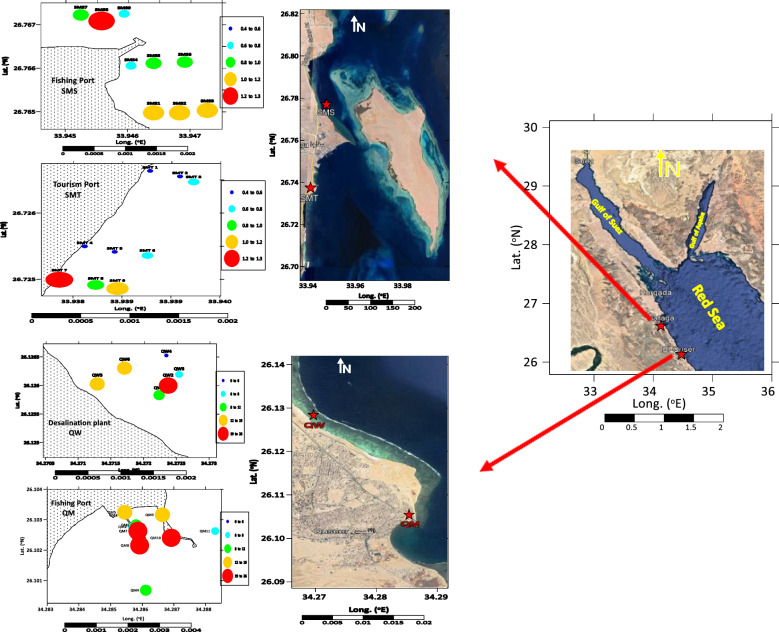
Illustrated in legend, circles with varying radiuses represent different concentration classes), El-Qusier, Red Sea coast, Egypt

The present study concerns two main cities on the Egyptian Red Sea coast, including 35 sampling sites totaling 90.3 km, extending from El-Qusier City to Safaga City (Fig. 1).

El-Quseir City is located 135 km south of Hurghada. It is a tourist city where scuba diving and snorkeling are popular. El-Quseir is situated in the eastern region of Egypt, along the coastline of the Red Sea. In the past, the location served as the terminus of the Wadi Hammamat trail, a significant pathway that linked Egypt with the Red Sea. El-Quseir is 130 km north of Marsa Alam, and 138 km south of Hurghada [41]. This area includes two sectors, sector 1 and 2. Within these sectors are many establishments, including the Fishing Port (QM), with 11 stations, and the Desalination Company (QW), with 8 stations.

Safaga City is located 60 km south of Hurghada. It is situated on the western coast of the Red Sea, about 50 km south of Hurghada City. It is a tourist and industrial area on the Red Sea coast, where it has several tourist resorts, a marine port, and a phosphate port. Safaga comprises sectors 3 and 4, consisting of the Tourist Port (SMT) with 9 stations and the Fishing Port (SMS) with an additional 9 stations. Additionally, Safaga Bay covers many subtidal habitats, such as seagrass meadows, coral-infused sand, mud, hardgrounds, and mangroves. The region comprises various human activities, including mining operations, marine and, tourism-related pursuits and businesses, shipping endeavors, fishing activities, and ship servicing facilities [41].

### Sampling collection and analysis

Seventy sediment samples were collected from 35 sampling stations during 2021 based on their ability to cover areas affected by industrial and human activity along the Egyptian Red Sea coast from lat. 26° 06′ 11.7″ N, long. 34° 17′ 7.3″ E to lat. 26° 46′ 2.1″ N, long. 33° 56′ 45.4″ E, whereas two surface sediment samples were collected from each site. At varying depths from 0 to 5 cm, a grab sampler was utilized from the sandy intertidal and back reef regions. The sample collection rigorously examined conventional methodologies, according to the International Atomic Energy Agency [26]. The specimens were first exposed to a controlled drying procedure in an oven for 48 h at 60 °C. The samples were homogenized using an agate pestle and mortar to achieve uniformity and then sieved through a 63 µm mesh sieve. To extract and analyze the heavy metals in the fine sediment, 0.5 g of the sediment powder was meticulously digested at a temperature of 85 °C. For this digestion process, a solution of HNO_3_ and HClO_4_ (3:1 v/v) was used, as described in Chester et al. [12]; Egbueri et al. [13], and [4]). The heavy metal analysis was conducted using the Perkin Elmer Analyst 100 atomic absorption spectrometer. The study was conducted using external calibration standards as references, confirming the correctness of the results. Three replicates were conducted for each heavy metal to improve accuracy, and the resultant data were then averaged to get the final reported values. The results were reported in µg/g.

#### Quality control

To achieve optimal accuracy in our study, we used chemicals that adhered to the highest purity requirements, as specified by analytical grade. The aqueous solutions used in this study were produced using deionized distilled water. Before use, all glassware and plastic apparatus underwent a comprehensive immersion process in a 10% nitric acid solution for an extended duration, often overnight. The detection limits were determined with a confidence level of 98%, equivalent to three standard deviations. It is worth noting that the accuracy of the measurements for these metals remained continuously within an acceptable range of 7.4% to 14.6%.

### Pollution assessment

Several environmental indices were utilized in the assessment of probable contaminants in the research region, depending on the concentrations of HM in shale rocks as the following indexes [22]:

#### Metal pollution index (MPI)

The Metal Pollution Index (MPI) is a statistic that considers the possible cumulative impact of heavy metals on human health. This evaluation aimed to examine the pollution in the area. The MPI was used to determine how concentrated all the parts being looked into were in the chosen areas and to put the sites into groups based on how polluted they were. Jahan and Strezov [27] conducted the estimation of MPI using Eq. (3):

1 $$\mathrm{MPI }= ({\text{M}}1\mathrm{ \times M}2\mathrm{ \times M}3\dots\mathrm{ Mn}) 1/\mathrm{n }$$

where n is the number of metals and M is the metal concentration µg/g. MPI value < 1 indicates the absence of contamination, whereas MPI value > 1 confirmation the presence of heavy metal pollution [60]

#### Factor of contamination

The contamination factor (CF) was created and can quantify contamination by [52]. The concentration factor (CF) is calculated using the given Equation by dividing the concentration of each metal in the sediments by the baseline or background value:

2 $$\mathrm{Contamination /Factor }=\mathrm{ C/ metal }/\mathrm{ C /background}$$

According to Håkanson (1980) and Han et al. [24], a CF value < 1 signifies a state of low pollution, while 1 < CF < 3 indicates moderate pollution. Furthermore, 3 < CF < 6 represents considerable pollution, and a CF > 6 signifies a state of very high pollution.

#### Contamination degree (CD)

The degree of contamination (Dc), derived as the sum of all contamination factors, may be used to quantify contamination at a given site. This index can be derived from the C_*F*_ values proposed by [22]:

3 $${\text{DC}}={\sum }_{{\text{i}}=1}^{{\text{n}}}{\text{CF}}$$

CF is the contamination factor, which measures the level of contamination in the number of heavy metals investigated. According to the given criteria, DC < 6 indicates a low level of contamination. In contrast, 6 < DC < 12 suggests a moderate level of contamination. 12 < DC < 24 signifies a significant level of contamination. However, DC > 24 indicates a very high level of contamination.

#### Pollution load index (PLI)

The Pollution Load Index (PLI) is a quantitative metric that quantifies how much a sample's metal content exceeds the background concentration. Jewel et al. [28] comprehensively assessed the extent of heavy metal toxicity in a specific sample. The contamination factor (CF) is vital in calculating the PLI index, acting as an essential component inside its formula [10].

4 $$PLI = \left({Cf}_{M1}\text {Cf}_{M2}\text{Cf}_{M3}\text{Cf}_{M4}\text {CF}_{Mn }\right)1/n$$

PLI value ≤ 1 indicates the absence of contamination, whereas PLI value > 1 confirmation the presence of heavy metal pollution [3]

### Risks assessment

#### Potential ecological risk (PEri)

[25] introduce the potential ecological risk index (PEri). This method has received widespread acceptance in the scientific community for assessing the harmful impacts of heavy metals in sedimentary settings [39].

5 $${{\text{E}}}_{{\text{f}}}^{{\text{i}}}= {{\text{C}}}_{{\text{f}}}^{{\text{i}}} \times {{\text{T}}}_{{\text{f}}}^{{\text{i}}}$$

${C}_{f}^{i}$ represents the level of pollution associated with a particular heavy metal at a specific place. It serves as an indicator of the pollution characteristics of the examined area, but it does not provide information on the environmental effects and risks resulting from this pollution. ${{\text{T}}}_{{\text{f}}}^{{\text{i}}}$ is the toxicity response coefficient pertains to an individual heavy metal. The study by Guo et al. [21] reported the following values: Cd = 30, Cu = Pb = Ni = 5, and Zn = 1. The present study aims to determine the index of possible toxicity response for various heavy metals in sediments, explicitly focusing on the Risk Index (RI). According to Liu et al. [37], the formula for calculating several heavy metals’ risk index (RI) is as follows:

6 $${\text{RI}}=\sum {{\text{E}}}_{{\text{f}}}^{{\text{i}}}$$

Hakanson [22] established a classification system for classifying the possible ecological danger associated with different metals. The grading requirements are as follows: a value of Eri < 40 indicates a low risk,a value of ${40 \le E}_{f}^{i}$ < 80 indicates a moderate risk; a value of ${80 \le E}_{f}^{i}$ < 160 indicates a considerable risk, a value of ${160 \le E}_{f}^{i}$ < 320indicates a high risk, and lastly, a value of 320 ≤ ${E}_{f}^{i}$ indicates a very high risk. The grading requirements for assessing the possible ecological danger of heavy metals are as follows: (RI) < 150 is classified as Low grade, a value between 150 ≤ RI < 300 falls into the Moderate category, a value between 300 ≤ RI < 600 is considered High, and a value > 600 is classified as Very High.

#### Contamination security index (CSI)

The Contamination Severity Index (CSI) is an indicator that was introduced by [45]. It is designed to measure the ecological risk associated with heavy metal pollution in sediment. Despite being a relatively recent indicator, CSI has significant value in academic discourse. (CSI) was computed using Eq. (7):

7 $${\text{CSI}}={\sum }_{i=1}^{n}Wt \left({\left(\frac{{\text{Csi}}}{{\text{ERLi}}}\right)}^{1/2}+{\left(\frac{{\text{Csi}}}{{\text{ERMi}}}\right)}^{2}\right)$$

C_Si_ represents the metal concentration, n is the number of analyzed hazardous elements, Wt is the calculated weight of each component (0.25, 0.134, 0.075, 0.251, 0.215, and 0.075), ERM_i_ is the effects range median (9.6, 370, 270, 218, 51.6, and 410).

The study conducted by Pejman et al. [45] revealed various effects for several heavy metals. Specifically, the effects ranged from 1.2 for Cd, 81 for Cr, 34 for Cu, 46.7 for Pb, 20.9 for Ni, and 150 for Zn. If the value of CSI is less than 0.5, it may be inferred that the sample is uncontaminated. The value of CSI, which falls between the 0.5 < CSI < 1, is relatively low. CSI value between 1 < CSI < 1.5 may be classified as low, while a value between 1.5 < CSI < 2 can be considered low to moderate. CSI value between 2 < CSI < 2.5 falls into the moderate range, while a value between 2.5 < CSI < 3 can be categorized as moderate to high. The value of CSI falls within the high range, namely between 3 < CSI < 4. The value of CSI falls between the range of 4 < CSI < 5, indicating a very high level. Furthermore, when CSI > 5, it may be classified as ultra-high.

#### Anthropogenesis method

Anthropogenicity (Apn%) is a quantitative assessment of the proportionate influence of human activities on metal concentration levels. Properly assessing heavy metal pollution in the sedimentary layers of Egypt's Red Sea is a critical problem for efficiently managing marine ecosystems. It is premeditated as the following Eq. (8):

8 $$\mathrm{Apn \% }=\frac{\upmu }{{\text{Bn}}}\mathrm{ \times }100$$

where: μ = determined concentration, whereas Bn = background value.

### Human health risk assessment

Risk assessment is a systematic technique that detects, defines, and assesses hazardous items to evaluate their possible negative repercussions over time. Additionally, USEPA, 2012 can predict the potential health effects of exposure to carcinogenic and non-carcinogenic substances. Individuals who live near polluted aquatic habitats are at risk of heavy metal poisoning. Another critical risk assessment component is dose–response research, which aims to identify the toxicity associated with various chemical exposure levels (Hidayati et al., 2020). In this context, it is vital to include the reference dose (RfD), which acts as a level below which the substance does not cause cancer. The Cancer Slope Factor (CSF) Kamunda et al. [30] frequently measure a substance’s carcinogenic potential.

#### a-Non-carcinogenic effect or “Hazard Quotients” (HQ)

The numerical models used for non-cancer risk assessment were obtained from the USEPA, [53]. The quantification of non-carcinogenic risk is often accomplished via hazard quotient (HQ) and hazard index (HI). To find the Hazard Quotient (HQ) for a certain contaminant, divide the expected daily intake by the reference dose (RfD) for that contaminant [29, 30]. The calculations for exposures from eating and cutaneous contact were determined separately using Eqs. (9 and 10).

9 $$\mathrm{THQ\, ingestion}=\frac{CxIRsxEDxEF}{BwxATx RfD}$$

10 $$\mathrm{THQ\, Dermal}=\frac{CxCFx SAx AFx ABSx EFxETx ED}{BwxAT xRfD}$$

where IRs ingestion rate, ED exposure duration, C is the concentration of heavy metals in the sediments, EF exposure frequency, BW body weight, average time, and RfD reference dose, respectively (Additional file 1: Table S1).

According to Wang et al. [55], THQ value < 1 signifies the absence of detrimental impacts on human health over a lifetime. However, if the THQ > 1, it implies the possibility of a non-carcinogenic public health risk arising from exposure to heavy metals. Moreover, as the THQ value increases, the probability of such a hazard also escalates, indicating a higher likelihood of adverse health effects.

#### The hazard index (HI)

Calculating the cumulative hazard quotient (HQ) for each hazardous material exhibiting undesirable or comparable toxic effects might indicate the acceptability of the associated risk. The integration of hazard quotients (HQs) from all exposure paths is reported to have comparable toxic effects [54]. The hazard index (HI) was computed using the method shown in Eq. 11.

11 $${\text{HI}}= {{\text{THQ}}}_{{\text{ing}}}+ {{\text{THQ}}}_{{\text{derm}}}$$

According to USEPA, [53], the Hazard Index (HI) value is < 1. This value suggests that the potential harm posed by HI to human health is either insignificant or non-existent. On the other hand, if the Hazard Index (HI) > 1, several paths are deemed undesirable. This indicates that the population exposed to these pathways may encounter detrimental health consequences, necessitating the implementation of risk management strategies. According to Johnbull et al. [29], treatments exist to mitigate health risks, and it is essential to implement preventative measures in response to these risks.

#### a-Carcinogenic risk assessment

The possible cancer risk associated with heavy metals present in sediment was estimated using the incremental or excess individual lifetime cancer risk assessment method. The cancer slope factor (CSF) was used to quantify the conversion of heavy metal exposure during an individual's lifetime into the corresponding risk of acquiring cancer USEPA,[53]. Cancer risk was calculated for all matrices using Eq. (12).

12 $$\mathrm{Cancer Risk }= \sum \mathrm{EXP }\times \mathrm{ CSF}$$

CR (mg/kg/day)^−1^ is the carcinogenic slope factor USEPA, [53]. The slope factor transforms the expected daily intake of heavy metals, averaged across a person's lifetime of exposure, into the incremental risk of that individual acquiring cancer. When the concentration ratio (CR) surpasses the range of 1 × 10^–4^–1 × 10^–6^, further evaluation specific to the chemical is necessary. According to Johnbull et al. [29], if the CR value drops below the range of 1 × 10^–4^–1 × 10^–6^, there is no need for intervention in terms of human health.

### Data analysis

To examine the various associations between heavy metals in the sediment of the studied region, we computed the correlation coefficient matrix (r) using the statistical software SPSS (Version 20). A Pearson’s correlation coefficient matrix was computed to assess the presence of a linear connection among the items. Analytical blanks were supplied for all determinations. To guarantee the procedure’s correctness, reference material in the form of marine sediment was used. The metal analysis’s recovery results range from 75 to 81%.

## Results and discussion

### Heavy metals distribution

**Fig. 2 a:**
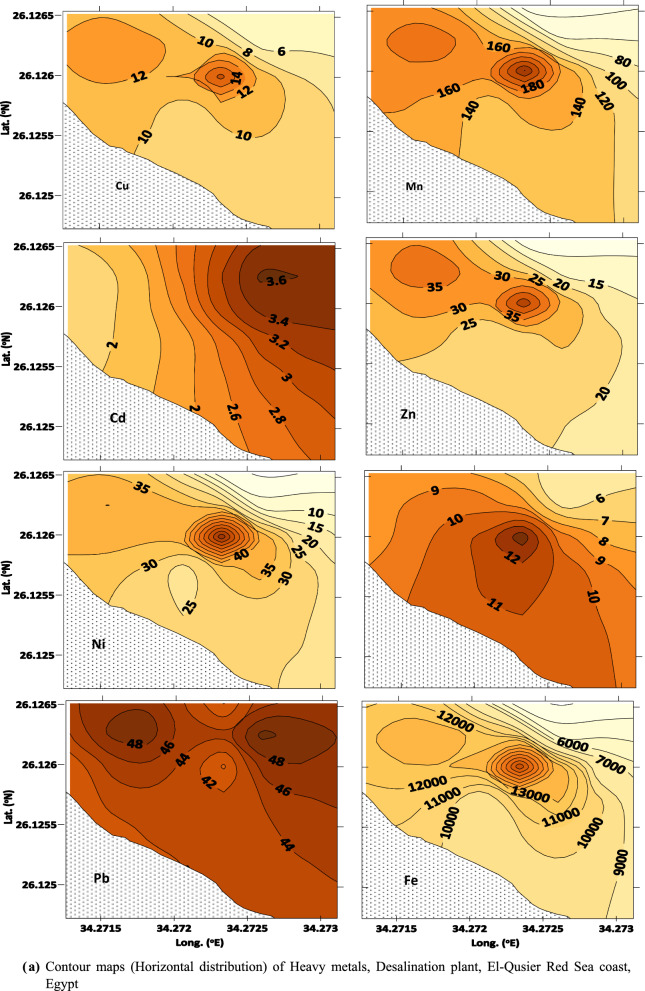

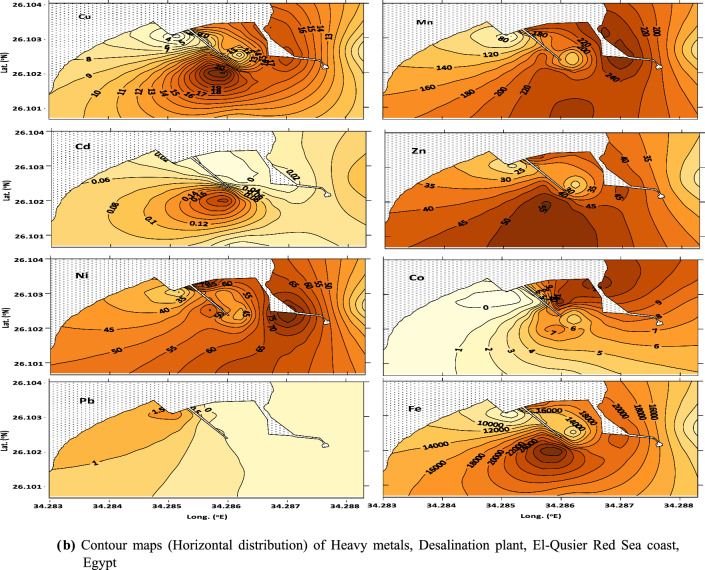
Contour maps (horizontal distribution) of Heavy metals, Desalination plant, El-Qusier Red Sea coast, Egypt. **b** Contour maps (horizontal distribution) of Heavy metals, Desalination plant, El-Qusier Red Sea coast, Egypt

**Fig. 3 a:**
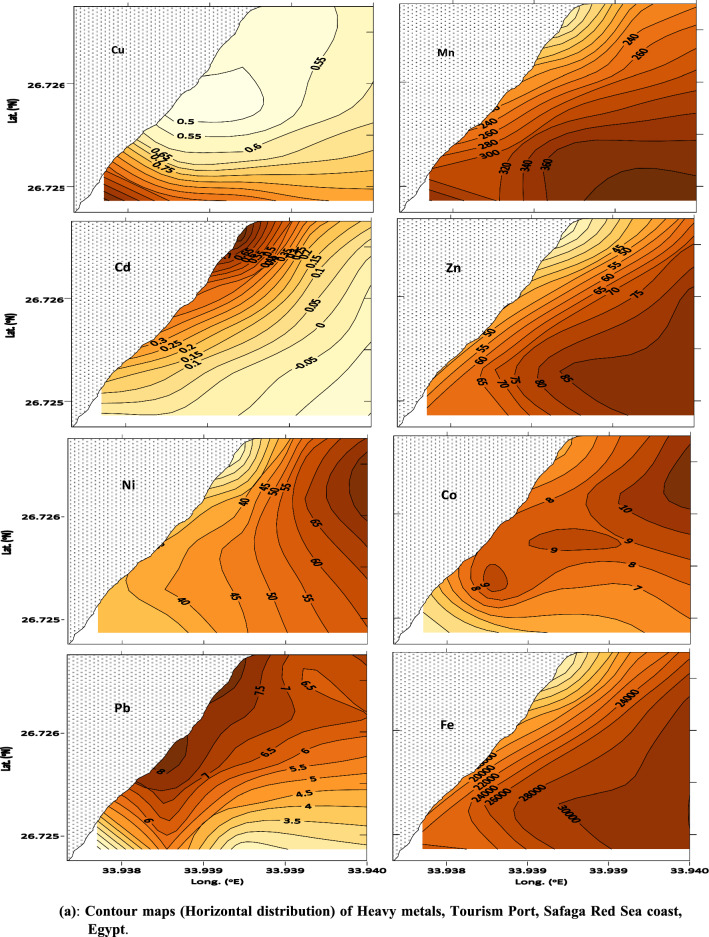

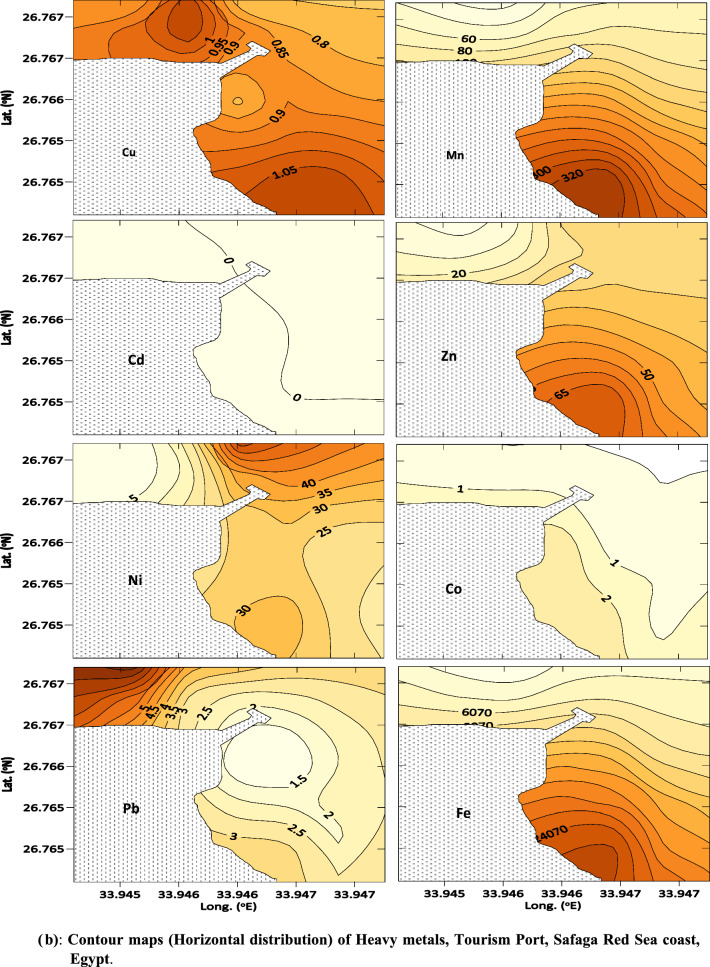
Contour maps (horizontal distribution) of Heavy metals, Tourism Port, Safaga Red Sea coast, Egypt. **b** Contour maps (horizontal distribution) of Heavy metals, Tourism Port, Safaga Red Sea coast, Egypt

Sediments play an important role in biogeochemical cycles; due to their ability to collect metals and other organic pollutants, their quality reflects marine environmental pollution. Heavy metal concentrations in sediment samples from El-Qusier and Safaga are summarized (Table 1, Figs. 2a,b and 3a,b). Heavy metal distribution in sediment samples differed across locations, sectors, and metals. The maximum values for the metals under consideration came in the following order: Fe (73.15–36296.44), Mn (9.42–380.24) > Zn (5.75–96.97) > Ni (1.49–91.43) > Pb (3.90–52.50) > Cu (0.97–39.87) > Co (1.17–15.17) > Cd (0.96–7.92) µg/g respectively. Fe and Mn recorded the highest concentrations, whereas Cd had the lowest. The higher metal concentrations found at different places were mainly linked to human activities such as marine ship paint, corrosion of maritime structures, landfilling, and building residue deposition [4, 40].

**Table 1:** Metal concentrations and Metal pollution index (MPI) in sediments of El-Qusier and Safaga sector

City	Sector	Stations	Cd, µg/g	Pb, µg/g	Ni, µg/g	Co, µg/g	Cu, µg/g	Zn, µg/g	Mn, µg/g	Fe, µg/g	MPI, µg/g
El-Quseir	Fishing port (sector 1)	Q M1	1.21	18.80	18.22	9.12	0.97	13.66	41.78	1539.31	15.43
		Q M2	1.96	13.00	56.06	5.65	7.51	32.43	153.53	11,303.95	36.86
		Q M3	1.12	18.90	17.67	5.23	6.76	7.39	23.06	844.61	14.45
		Q M4	2.01	18.30	67.90	2.55	10.84	53.05	246.63	17,032.79	44.49
		Q M5	1.93	16.90	87.66	5.76	15.11	52.70	249.95	24,171.14	54.62
		Q M6	2.45	22.60	15.18	11.35	9.02	21.84	134.97	7740.95	34.41
		Q M7	0.96	19.60	75.65	9.23	19.34	53.46	294.01	24,096.84	55.96
		Q M8	2.71	25.11	91.43	15.00	26.00	56.91	296.30	35,047.18	78.45
		Q M9	2.06	18.10	68.11	12.50	18.49	42.86	265.99	21,052.58	58.69
		Q M10	2.31	14.90	75.43	8.77	19.71	48.59	235.49	24,565.42	57.89
		Q M11	1.03	21.00	20.12	7.22	6.68	16.77	98.10	6421.62	26.19
	Desalination company (sector 2)	QW 1	2.46	42.80	19.01	11.49	8.34	17.06	117.50	7877.85	36.32
		QW 2	3.22	38.90	89.51	14.16	20.55	53.70	287.50	28,366.10	78.41
		QW 3	3.68	52.50	10.99	5.88	6.25	16.11	75.63	5279.81	29.01
		QW 4	3.07	39.80	1.49	6.30	4.57	6.27	32.54	1742.27	14.41
		QW 5	1.86	40.50	36.27	8.29	11.91	28.22	165.69	12,284.84	44.54
		QW 6	2.00	51.50	42.55	9.49	13.51	41.94	202.58	14,816.81	53.85
Average El-Quseir city*			**2.12 ± 0.78**	**27.84 ± 13.28**	**46.66 ± 31.36**	**8.70 ± 3.39**	**12.09 ± 6.83**	**33.11 ± 18.24**	**171.84 ± 95.91**	**14,363.77 ± 10,376.28**	**43.18 ± 20.05**
Safaga City	Tourist port (sector 3)	SMT1	6.10	17.20	7.87	5.57	7.02	16.26	72.08	4985.72	25.68
		SMT2	7.92	23.50	84.10	7.66	14.27	36.08	229.34	17,802.13	63.12
		SMT3	4.85	7.10	54.06	12.84	32.50	77.55	252.77	29,930.39	67.97
		SMT4	5.89	7.90	34.05	5.17	10.49	28.88	193.49	11,796.10	39.27
		SMT5	4.44	5.40	49.02	15.17	39.87	96.97	380.24	36,296.44	74.52
		SMT6	6.21	14.30	45.21	6.01	29.46	84.71	350.50	29,043.88	70.54
		SMT7	5.75	12.80	33.03	2.74	24.20	64.27	369.31	25,321.87	56.01
		SMT8	6.06	12.50	28.61	3.76	20.07	58.80	298.55	22,425.62	53.22
		SMT9	5.97	5.20	37.85	4.67	26.34	68.79	352.76	25,737.62	55.52
	Fishing port (sector 4)	SMS1	6.45	12.50	35.74	2.73	25.99	75.12	364.55	28,841.79	59.71
		SMS2	5.46	7.60	22.95	1.17	14.22	49.11	239.69	17,567.34	36.66
		SMS3	6.11	3.90	15.70	1.67	15.66	42.67	210.52	16,239.51	33.06
		SMS4	4.98	12.40	27.26	2.72	11.86	43.30	187.07	15,559.01	40.21
		SMS5	6.18	13.00	28.45	2.23	11.41	38.96	177.25	14,287.95	39.35
		SMS6	6.80	8.90	19.94	2.77	7.00	35.85	113.21	7449.71	30.29
		SMS7	4.88	12.50	15.60	2.26	1.42	5.75	9.42	335.12	9.28
		SMS8	5.49	17.40	6.00	2.20	1.59	9.53	11.14	73.15	7.93
		SMS9	5.26	4.90	65.37	1.96	12.84	30.33	46.29	2128.53	24.42
Average Safaga city**			**5.82 ± 0.81**	**11.06 ± 5.18**	**33.93 ± 20.05**	**4.63 ± 3.84**	**17.01 ± 10.74**	**47.94 ± 26.06**	**214.34 ± 125.04**	**16,990.10 ± 11,005.00**	**43.71 ± 20.04**
Average Red sea***			**4.02 ± 2.03**	**19.21 ± 12.99**	**40.12 ± 24.56**	**6.61 ± 4.13**	**14.62 ± 9.27**	**40.74 ± 23.51**	**193.70z ± 112.30**	**15,714.45 ± 10,629.94**	**43.45 ± 19.75**

*Average concentration for El-Qusier city

**Average concentration for Safaga city

***Average concentration for Red Sea (El-Qusier and Sagfaga)

El-Qusier City is extremely severe with Cd and very severely enriched with Pb. Stations QM8 in Sector 1 (fishing port) and QW3 in Sector 2 have the most significant concentrations of almost all metals. This may be because fishing boats need constant maintenance and repair, which could entail using heavy metals. In addition, desalination plants increase the concentration of heavy metals in saltwater outflow, which can harm marine animals and ecosystems. Moreover, the El-Quseir area is related to the phosphate shipping movement, fishing boats, and the tourism industry. These activities may increase heavy metal concentrations in the surrounding environment via various methods [41].

In contrast, overall heavy metal concentrations in Safaga City exhibited a consistent upward trend at stations SMT2 and SMT5 in the tourist port (Table 1). These findings could be attributed to port activity, as evidenced by the high volume of boat traffic in tourism ports, which can cause increased sediment disturbance and the release of heavy metals that have accumulated in sediments over time, as well as the discharge of wastewater containing heavy metals into nearby waters, where the heavy metals can accumulate in sediments and be taken up by aquatic organisms [41]. The research identified industrial and household wastewater outflow, maritime operations, and agricultural runoff as anthropogenic drivers of heavy metal contamination in the bay. According to the study, the bay's sediments were enriched with heavy metals compared to background values, which could harm the environment and human health. According [14], the growing Mn concentration in northern Red Sea offshore sediments is due to its absorption into the calcite crystal lattice. The rise in Zn levels in Safaga Bay might be linked to mineral commodity transportation, including zinc and phosphate, and mining operations in the eastern desert [14, 15].

Finally, Table 2 compares heavy metal concentrations in sediment samples from Egypt's Red Sea Coast to other comparable places in Egypt and across the world. The results demonstrated that the Egyptian Red Sea shoreline had a greater concentration of Cd than the surrounding shale, continental crust, and SQGs. Furthermore, Pb concentrations in the examined region are more significant than in background shale and continental crust but lower than in SQGs, indicating that Pb may occasionally have deleterious biological consequences [33]. The Ni and Cu concentrations are more significant than in SQGs. However, the two metal concentrations are lower than the shale and continental crust background levels. Some coastal areas, like south Safaga Bay, do have weathering effects in the form of phosphate and basement fragments, but the main culprits when it comes to trace metals are landfills, plastic waste, phosphate operations, fishing vessels, shipyards, people, and tourist activities [41]. This result suggested that these metals may have descended from the exact origins. Furthermore, it exhibits similar migratory and transportation activities under the same environmental circumstances [55].

**Table 2:** Comparison of heavy metals concentration in marine sediments in various regions in Red Sea Coast

Location	Fe	Pb	Co	Ni	Zn	Cd	Mn	Cu	Reference
Red Sea coast	14.562.50	4.89	4.81	15.37	27.55	–	291.94	7.70	[17]
Red Sea coast (Hurghada)	355.44	42.38	1.66	1.74	7.77	0.14	51.95	1.26	[42]
Yemen (Red sea)	–	6.47	–	8.98	36.81	0.76	–	17.34	[2]
Saudi Arabia (gulf of Aqab)	3374	6.6	4.5	14	24	0.91	184	3074	[16]
Red Sea coast (Shalateen)	–	3.76	2.59	10.19	25.17	0.39	–	4.17	[50]
Red Sea coast	2923.85	4.06	4.45	18.42	29.10	0.16	145.85	2.51	[18]
Saudi Arabia (Jeddah)	–	77.34	–	3.68	18.02	80	36.52	9.18	Halawani, et al., [23]
Red Sea coast (Al-Quseir)	14,363.77	27.84	8.70	46.66	33.11	2.12	171.84	12.09	Present study
Red Sea coast (Safaga)	16,990.10	11.06	4.63	33.93	47.94	5.82	214.34	17.01	
SQGs (ERL)	–	46.7	–	20.90	15	1.20	–	34.0	Long et al. [38]
SQGs (LER)	4000	31.0	–	16	120	0.6	46.0	16.00	

### Pollution assessment

The assessment of heavy metal pollution in sediments is currently conducted using various sediment pollution indices, including MPI, CF, CSI, and PLI, as well as normalization techniques, and by comparing the results with sediment quality guidelines and regulations [32, 36].

#### Metal pollution index (MPI)

The Metal Contamination Index (MPI) is intended to measure metal contamination without regard to the influence of specific metals. Metals discovered in the sediments of El-Quseir and Safaga show variable degrees of contamination for diverse purposes. The authorized limits of trace metals established by [11] for aquatic life protection were used to calculate the maximum acceptable concentration (MAC).

The MPI findings (Table 2) revealed that station QM8 in Sector 1 was the most polluted station in El-Quseir City. This was due to the regions having directly or indirectly endured significant environmental stress due to numerous discharges and fishing port activities. Meanwhile, in the Safaga area, station SMT5 was revealed to have the highest MPI. Safaga has higher mean values of virtually all metals than El-Quseir, except Pb, Ni, and Co, which have higher mean levels in El-Quseir. Overall, human activities such as industrial and household wastewater discharge, marine activities, and agricultural runoff impact the distribution and enrichment of heavy metals in Safaga Bay, Egypt's recent sediments. These results highlight the need for proper management measures to limit and avoid heavy metal pollution in Safaga Bay [14].

#### Contamination factor CF

According to Table 3, the CF values of individual metals < 1 in the majority of the tested sites indicate a low degree of contamination except for Cd, which ranged from (0.2–26.40) with an average mean value (12.84) indicating a range between low and moderate contamination at El-Qusier City and a range between moderate and very high contamination with an average degree of considerable contamination at Safaga City. Stations QW2 and SMT2 were identified as having severely contaminated sediment from the petroleum and phosphate sectors and several natural and human causes. Human activities such as operating tourist boats, scuba diving, plastic garbage, and gasoline combustion all influence the area [4].

**Table 3:** Contamination factor (CF) and contamination degree (Cdeg), values in El-Qusier and Safaga sectors during 2021

City	Sector	Stations	Cd	Pb	Ni	Co	Cu	Zn	PLI	CD	Degree
			CF								
El Qusier	Fishing port	Q M1	4.03	0.94	0.27	0.48	0.02	0.14	0.34	5.89	Low
		Q M2	6.53	0.65	0.82	0.30	0.17	0.34	0.62	8.81	Moderated
		Q M3	3.73	0.95	0.26	0.28	0.15	0.08	0.38	5.44	Low
		Q M4	6.70	0.92	1.00	0.13	0.24	0.56	0.69	9.55	Moderated
		Q M5	6.43	0.85	1.29	0.30	0.34	0.55	0.86	9.76	Moderated
		Q M6	8.17	1.13	0.22	0.60	0.20	0.23	0.62	10.55	Moderated
		Q M7	3.20	0.98	1.11	0.49	0.43	0.56	0.86	6.77	Moderated
		Q M8	9.03	1.26	1.34	0.79	0.58	0.60	1.27	13.60	Considerable
		Q M9	6.87	0.91	1.00	0.66	0.41	0.45	0.96	10.29	Moderated
		Q M10	7.70	0.75	1.11	0.46	0.44	0.51	0.93	10.97	Moderated
		Q M11	3.43	1.05	0.30	0.38	0.15	0.18	0.47	5.48	Low
	Desalination company	QW 1	8.20	2.14	0.28	0.60	0.19	0.18	0.68	11.59	Moderated
		QW 2	10.73	1.95	1.32	0.75	0.46	0.57	1.32	15.76	Considerable
		QW 3	12.27	2.63	0.16	0.31	0.14	0.17	0.58	15.67	Considerable
		QW 4	10.23	1.99	0.02	0.33	0.10	0.07	0.32	12.74	Considerable
		QW 5	6.20	2.03	0.53	0.44	0.26	0.30	0.78	9.76	Moderated
		QW 6	6.67	2.58	0.63	0.50	0.30	0.44	0.94	11.11	Moderated
Safag city	Tourist port	SMT1	20.33	0.86	0.12	0.29	0.16	0.17	0.50	21.93	V. High
		SMT2	26.40	1.18	1.24	0.40	0.32	0.38	1.11	29.91	Considerable
		SMT3	16.17	0.36	0.80	0.68	0.72	0.82	1.10	19.53	Considerable
		SMT4	19.63	0.40	0.50	0.27	0.23	0.30	0.65	21.34	Considerable
		SMT5	14.80	0.27	0.72	0.80	0.89	1.02	1.13	18.50	Considerable
		SMT6	20.70	0.72	0.66	0.32	0.65	0.89	1.10	23.94	Considerable
		SMT7	19.17	0.64	0.49	0.14	0.54	0.68	0.82	21.65	Considerable
		SMT8	0.20	0.63	0.42	0.20	0.45	0.62	0.38	2.51	Low
		SMT9	19.90	0.26	0.56	0.25	0.59	0.72	0.82	22.27	Considerable
	Fishing port	SMS1	21.50	0.63	0.53	0.14	0.58	0.79	0.88	24.16	V. High
		SMS2	18.20	0.38	0.34	0.06	0.32	0.52	0.53	19.81	Considerable
		SMS3	20.37	0.20	0.23	0.09	0.35	0.45	0.48	21.68	Considerable
		SMS4	16.60	0.62	0.40	0.14	0.26	0.46	0.64	18.48	Considerable
		SMS5	20.60	0.65	0.42	0.12	0.25	0.41	0.64	22.45	Considerable
		SMS6	22.67	0.45	0.29	0.15	0.16	0.38	0.54	24.08	V. High
		SMS7	16.27	0.63	0.23	0.12	0.03	0.06	0.28	17.33	Considerable
		SMS8	18.30	0.87	0.09	0.12	0.04	0.10	0.29	19.51	Considerable
		SMS9	17.53	0.25	0.96	0.10	0.29	0.32	0.58	19.45	Considerable

#### Contamination severity index (CSI)

The results shown in Table 4 show that the average CSI values in the El-Quseir and Safaga sectors were clean for the elements that were looked at. The average CSI values for sample sites in El-Qusier varied from unconsummated to considerably low, whereas Safaga varied from low to moderate. Based on the results of this new index, the lowest Contamination Severity Index (CSI) value seen in the studied area suggests a relatively low level of contamination. When the indicators' precise results are examined, the estimated pollution level index (PLI) is lower than the comprehensive pollution status index (CSI) in the chosen research region. The primary factor contributing to this disparity is the need for a comprehensive understanding of the research region's history in the field of study. The outcomes derived from the indices above are primarily based on shale values, which do not accurately reflect the background content of the study area.

**Table 4:** Potential ecological risk index (RI) and Contamination Security Index of El-Qusier and Safaga sectors during 2021

		Eri						RI		CSI	
			Cd	Pb	Ni	Cu	Zn	RI	Degree	CSI	Degree
El Quseir	Fishing port	Q M1	121.00	4.70	1.34	0.11	0.14	127.29	Low	0.68	Very low
		Q M2	196.00	3.25	4.12	0.83	0.34	204.55	Moderate	1.14	Low
		Q M3	112.00	4.73	1.30	0.75	0.08	118.85	Low	0.68	Very low
		Q M4	201.00	4.58	4.99	1.20	0.56	212.33	Moderate	1.34	Low
		Q M5	193.00	4.23	6.45	1.68	0.55	205.90		1.64	Considerable low
		Q M6	245.00	5.65	1.12	1.00	0.23	253.00		0.82	Very low
		Q M7	96.00	4.90	5.56	2.15	0.56	109.17	Low	1.37	Low
		Q M8	271.00	6.28	6.72	2.89	0.60	287.49	Moderate	1.82	Considerable low
		Q M9	206.00	4.53	5.01	2.05	0.45	218.04		1.36	Low
		Q M10	231.00	3.73	5.55	2.19	0.51	242.97		1.47	Low
		Q M11	103.00	5.25	1.48	0.74	0.18	110.65	Low	0.71	Very low
	Desalination company	QW 1	246.00	10.70	1.40	0.93	0.18	259.20	Moderate	0.92	Very low
		QW 2	322.00	9.73	6.58	2.28	0.57	341.15	High	1.87	Considerable low
		QW 3	368.00	13.13	0.81	0.69	0.17	382.80		0.98	Very low
		QW 4	307.00	9.95	0.11	0.51	0.07	317.63		0.77	
		QW 5	186.00	10.13	2.67	1.32	0.30	200.41	Moderate	1.03	Low
		QW 6	200.00	12.88	3.13	1.50	0.44	217.95		1.15	
Safaga	Tourist port	SMT1	610.00	4.30	0.58	0.78	0.17	615.83	V. high	1.01	
		SMT2	792.00	5.88	6.18	1.59	0.38	806.02		2.08	Moderate
		SMT3	485.00	1.78	3.98	3.61	0.82	495.18	High	1.38	Low
		SMT4	589.00	1.98	2.50	1.17	0.30	594.95		1.20	
		SMT5	444.00	1.35	3.60	4.43	1.02	454.40		1.29	
		SMT6	621.00	3.58	3.32	3.27	0.89	632.06	V. high	1.43	
		SMT7	575.00	3.20	2.43	2.69	0.68	583.99	High	1.24	
		SMT8	606.00	3.13	2.10	2.23	0.62	614.08	V. high	1.22	
		SMT9	597.00	1.30	2.78	2.93	0.72	604.73		1.26	
	Fishing port	SMS1	645.00	3.13	2.63	2.89	0.79	654.43		1.33	
		SMS2	546.00	1.90	1.69	1.58	0.52	551.68	High	1.08	
		SMS3	611.00	0.98	1.15	1.74	0.45	615.32	V. high	1.04	
		SMS4	498.00	3.10	2.00	1.32	0.46	504.88	High	1.10	
		SMS5	618.00	3.25	2.09	1.27	0.41	625.02	V. high	1.20	
		SMS6	680.00	2.23	1.47	0.78	0.38	684.85		1.15	
		SMS7	488.00	3.13	1.15	0.16	0.06	492.49	High	0.94	Very low
		SMS8	549.00	4.35	0.44	0.18	0.10	554.07		1.01	Low
		SMS9	526.00	1.23	4.81	1.43	0.32	533.78		2.08	Moderate

A comparison was conducted between the data obtained from the PLI index and a newly developed index, revealing that the latter exhibits much higher sensitivity. Overall, the outcomes produced by the new measure in this study are much more dependable and coherent than the previous indices. While the CSI does not have any baseline values and does include site-specific factors, it is a better and more accurate way to describe heavy metal contamination in water than other methods [45]. Nevertheless, it is essential to note that previously published findings may exhibit modest variations due to disparities in the locations where samples were collected and the analytical techniques used.

#### Pollution load index PLI

In contrast, the Contamination Factor (CF) is an essential component of the PLI index's formula and plays a vital role in its calculation [10]. When the geographical distribution of PLI values within the research region is examined, it is clear that they varied from 0.28 to 1.32 (Table 3). Because all PLI values < 1 are lower than the standard level, this range indicates the absence of heavy metal contamination. This observation applies to all Red Sea monitoring sites. As a result, it can be safely said that the sediment samples show no contamination in terms of PLI values [28]. Specifically, El-Quseir City (0.74) > Safaga City (0.69), indicating that all areas are not contaminated.

#### Anthropogenic index (Apn%)

**Fig. 4:**
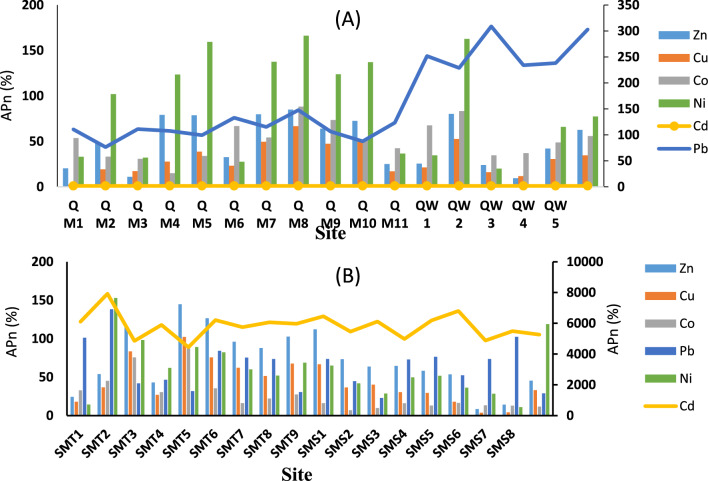
Anthropogenic percentage (Apn%) for influenced heavy metals of sediments in El-Quseir (**A**) and Safaga (**B**) sectors

Figure 4 depicts the anthropogenic origin of heavy metals in sediment samples from El-Quseir and Safaga. El-Quseir sector has the most significant relative quantities of Cd, Zn, Pb, Ni, Co, and Cu. In contrast, the Safaga area had the highest relative concentrations of Cd, Pb, Ni, Co, Zn, and Cu. Cd metal had the most significant influence in the El-Quseir and Safaga areas. According to the data, the sites QW 7 and SMT5 in the El-Quseir and Safaga sectors had the lowest Apn% for Cd, respectively.

In contrast, the Apn% values for Cd were most significant at QM3 and SMT2. The introduction of heavy metals through human activities has posed a significant ecological risk to species regarding speciation, with a particular emphasis on cadmium [35]. Cadmium is a chemical considered more poisonous than arsenic, and lead poses a significant ecological concern and significantly impacts the rates of poisoning reactions [58].

### Risks assessment

#### Potential ecological risk

The Potential Risk Index Method (PEri) is commonly used to assess the damage caused by heavy metals in sediments. The presence of five heavy metals (Cd, Pb, Ni, Cu, and Zn) was used to calculate the Risk Index (RI). The Ecological Risk Index Eri^Me^ and RI findings for these heavy metals in the surface sediments of the study area are shown (Table 4).

The metals were rated in terms of possible ecological risk index, varying in concentration levels, as follows: Cd (96.00–792.00) > Pb (0.98–13.13) > Ni (0.11–6.72) > Cu (0.11–4.34) > Zn (0.06–1.02), with average values of 402.40 > 4.80 > 2.95 > 1.62, and 0.43, respectively. The findings suggest a low ecological risk level for each element (Eri^Me^ < 40), except Cd (160 < Eri^Cd^ < 320) in El-Qusier City. Sfaga City has an Eri^Cd^ score > 320, indicating extremely significant pollution. Cd concentrations varied significantly throughout the study area, ranging from low to severely polluted areas. This might be attributable to human factors such as phosphate loading, oil pollution, and tourism [7]. Cd provided the most substantial ecological danger of the heavy metals studied owing to its high toxicity factor and propensity for long-term accumulation in the human body, leading to health difficulties such as renal dysfunction and reproductive deficits [57]. This significant ecological risk linked with Cd is consistent with Hakanson's approach, emphasizing its hazardous potential [57]. The findings of the RI ecological risk indexes corresponded to those reported by [18], and [15] on the Egyptian Red Sea coast,

Furthermore, the Risk Index (RI) values for surface sediments vary from 109.17 at station QM7 to 806.02 at station SMT2, with an average of 412.20. These findings led to the conclusion that heavy metals constitute a significant ecological danger to the surface sediments of the study region. El-Qusier’s RI readings in the 150 < RI < 300 range suggest a moderate ecological risk. At Safaga, RI values are > 300, indicating a high level of ecological danger.

Sedimentary heavy metals remain in the environment. Because metals in the sediment may be discharged into the overlying water, they can be hazardous to benthic creatures and aquatic organisms. When the quantities of certain minerals and essential elements in biology surpass particular levels, they destroy living creatures. Minerals connected with silt may accumulate in marine creatures’ tissues, negatively impacting the whole food chain. A tidal flat is an essential part of the coastal region’s hydrological and biological processes, and it is also great for animals, fishing, and enjoyment [56].

#### Human health risk assessment

Humans are exposed to carcinogenic and non-carcinogenic hazards via oral ingestion, inhalation, and skin contact. To find out how dangerous something is to people's health, the hazard index (HI) and lifetime carcinogenic risk (LCR) were used (Tables 6 and 7). This is calculated as chronic daily exposure. This study figured out the level of risk for high concentrations of the heavy metals that were studied by calculating the hazard index that would be caused by eating or touching the area that was being studied [54, 59]. The hazard index of non-carcinogenic hazards was one in the evaluated region and the individual assessed places (Table 5).

**Table 5:** Hazard Quotients for non-carcinogenic Risks ingestion and dermal in area of investigation during 2021

			Cd	Pb	Cu	Zn	Mn	Fe	HI
El Qusier	Fishing port	Q M1	3.3E−03	6.1E−02	3.3E−05	3.1E−04	6.0E−02	3.0E−03	1.3E−01
		Q M2	5.4E−03	4.2E−02	2.6E−04	7.4E−04	2.2E−01	2.2E−02	2.9E−01
		Q M3	3.1E−03	6.2E−02	2.3E−04	1.7E−04	3.3E−02	1.7E−03	1.0E−01
		Q M4	5.5E−03	6.0E−02	3.7E−04	1.2E−03	3.5E−01	3.3E−02	4.5E−01
		Q M5	5.3E−03	5.5E−02	5.2E−04	1.2E−03	3.6E−01	4.7E−02	4.7E−01
		Q M6	6.7E−03	7.4E−02	3.1E−04	5.0E−04	1.9E−01	1.5E−02	2.9E−01
		Q M7	2.6E−03	6.4E−02	6.6E−04	1.2E−03	4.2E−01	4.7E−02	5.4E−01
		Q M8	7.4E−03	8.2E−02	8.9E−04	1.3E−03	4.2E−01	6.9E−02	5.8E−01
		Q M9	5.6E−03	5.9E−02	6.3E−04	9.8E−04	3.8E−01	4.1E−02	4.9E−01
		Q M10	6.3E−03	4.9E−02	6.8E−04	1.1E−03	3.4E−01	4.8E−02	4.4E−01
		Q M11	2.8E−03	6.8E−02	2.3E−04	3.8E−04	1.4E−01	1.3E−02	2.2E−01
	Desalination pompany	QW 1	6.7E−03	1.4E−01	2.9E−04	3.9E−04	1.7E−01	1.5E−02	3.3E−01
		QW 2	8.8E−03	1.3E−01	7.0E−04	1.2E−03	4.1E−01	5.6E−02	6.0E−01
		QW 3	1.0E−02	1.7E−01	2.1E−04	3.7E−04	1.1E−01	1.0E−02	3.0E−01
		QW 4	8.4E−03	1.3E−01	1.6E−04	1.4E−04	4.6E−02	3.4E−03	1.9E−01
		QW 5	5.1E−03	1.3E−01	4.1E−04	6.4E−04	2.4E−01	2.4E−02	4.0E−01
		QW 6	5.5E−03	1.7E−01	4.6E−04	9.6E−04	2.9E−01	2.9E−02	4.9E−01
Safaga	Tourist port	SMT1	1.7E−02	5.6E−02	2.4E−04	3.7E−04	1.0E−01	9.8E−03	1.9E−01
		SMT2	2.2E−02	7.7E−02	4.9E−04	8.2E−04	3.3E−01	3.5E−02	4.6E−01
		SMT3	1.3E−02	2.3E−02	1.1E−03	1.8E−03	3.6E−01	5.9E−02	4.6E−01
		SMT4	1.6E−02	2.6E−02	3.6E−04	6.6E−04	2.8E−01	2.3E−02	3.4E−01
		SMT5	1.2E−02	1.8E−02	1.4E−03	2.2E−03	5.4E−01	7.1E−02	6.5E−01
		SMT6	1.7E−02	4.7E−02	1.0E−03	1.9E−03	5.0E−01	5.7E−02	6.2E−01
		SMT7	1.6E−02	4.2E−02	8.3E−04	1.5E−03	5.3E−01	5.0E−02	6.4E−01
		SMT8	1.7E−02	4.1E−02	6.9E−04	1.3E−03	4.3E−01	4.4E−02	5.3E−01
		SMT9	1.6E−02	1.7E−02	9.0E−04	1.6E−03	5.0E−01	5.0E−02	5.9E−01
	Fishing port	SMS1	1.8E−02	4.1E−02	8.9E−04	1.7E−03	5.2E−01	5.6E−02	6.4E−01
		SMS2	1.5E−02	2.5E−02	4.9E−04	1.1E−03	3.4E−01	3.4E−02	4.2E−01
		SMS3	1.7E−02	1.3E−02	5.4E−04	9.7E−04	3.0E−01	3.2E−02	3.6E−01
		SMS4	1.4E−02	4.0E−02	4.1E−04	9.9E−04	2.7E−01	3.0E−02	3.5E−01
		SMS5	1.7E−02	4.2E−02	3.9E−04	8.9E−04	2.5E−01	2.8E−02	3.4E−01
		SMS6	1.9E−02	2.9E−02	2.4E−04	8.2E−04	1.6E−01	1.5E−02	2.2E−01
		SMS7	1.3E−02	4.1E−02	4.8E−05	1.3E−04	1.3E−02	6.6E−04	6.8E−02
		SMS8	1.5E−02	5.7E−02	5.4E−05	2.2E−04	1.6E−02	1.4E−04	8.8E−02
		SMS9	1.4E−02	1.6E−02	4.4E−04	6.9E−04	6.6E−02	4.2E−03	1.0E−01

Dermal										
			Cd	Pb	Ni	Cu	Zn	Mn	Fe	HI
El Qusier	Fishing port	Q M1	8.2E−04	1.8E−03	1.1E−03	1.8E−04	1.5E−05	5.9E−04	3.7E−03	8.3E−03
		Q M2	1.3E−03	1.3E−03	3.4E−03	1.4E−03	3.7E−05	2.2E−03	2.7E−02	3.7E−02
		Q M3	7.6E−04	1.8E−03	1.1E−03	1.2E−03	8.4E−06	3.3E−04	2.0E−03	7.3E-03
		Q M4	1.4E−03	1.8E−03	4.1E−03	2.0E−03	6.0E−05	3.5E−03	4.1E−02	5.4E−02
		Q M5	1.3E−03	1.6E−03	5.3E−03	2.8E−03	6.0E−05	3.5E−03	5.9E−02	7.3E−02
		Q M6	1.7E−03	2.2E−03	9.2E−04	1.7E−03	2.5E−05	1.9E−03	1.9E−02	2.7E−02
		Q M7	6.5E−04	1.9E−03	4.6E−03	3.6E−03	6.0E−05	4.2E−03	5.8E−02	7.3E−02
		Q M8	1.8E−03	2.4E−03	5.5E−03	4.8E−03	6.4E−05	4.2E−03	8.5E−02	1.0E−01
		Q M9	1.4E−03	1.8E−03	4.1E−03	3.4E−03	4.8E−05	3.8E−03	5.1E−02	6.6E−02
		Q M10	1.6E−03	1.4E−03	4.6E−03	3.6E−03	5.5E−05	3.3E−03	6.0E−02	7.4E−02
		Q M11	7.0E−04	2.0E−03	1.2E−03	1.2E−03	1.9E−05	1.4E−03	1.6E−02	2.2E−02
	Desalination company	QW 1	1.7E−03	4.1E−03	1.2E−03	1.5E−03	1.9E−05	1.7E−03	1.9E−02	2.9E−02
		QW 2	2.2E−03	3.8E−03	5.4E−03	3.8E−03	6.1E−05	4.1E−03	6.9E−02	8.8E−02
		QW 3	2.5E−03	5.1E−03	6.7E−04	1.2E−03	1.8E−05	1.1E−03	1.3E−02	2.3E−02
		QW 4	2.1E−03	3.9E−03	9.0E−05	8.4E−04	7.1E−06	4.6E−04	4.2E−03	1.2E−02
		QW 5	1.3E−03	3.9E−03	2.2E−03	2.2E−03	3.2E−05	2.3E−03	3.0E−02	4.2E−02
		QW 6	1.4E−03	5.0E−03	2.6E−03	2.5E−03	4.7E−05	2.9E−03	3.6E−02	5.0E−02
Safaga	Tourist port	SMT1	4.1E−03	1.7E−03	4.8E−04	1.3E−03	1.8E−05	1.0E−03	1.2E−02	2.1E−02
		SMT2	5.4E−03	2.3E−03	5.1E−03	2.6E−03	4.1E−05	3.2E−03	4.3E−02	6.2E−02
		SMT3	3.3E−03	6.9E−04	3.3E−03	6.0E−03	8.8E−05	3.6E−03	7.3E−02	8.9E−02
		SMT4	4.0E−03	7.7E−04	2.1E−03	1.9E−03	3.3E−05	2.7E−03	2.9E−02	4.0E−02
		SMT5	3.0E−03	5.2E−04	3.0E−03	7.4E−03	1.1E−04	5.4E−03	8.8E−02	1.1E−01
		SMT6	4.2E−03	1.4E−03	2.7E−03	5.4E−03	9.6E−05	5.0E−03	7.0E−02	8.9E−02
		SMT7	3.9E−03	1.2E−03	2.0E−03	4.5E−03	7.3E−05	5.2E−03	6.1E−02	7.8E−02
		SMT8	4.1E−03	1.2E−03	1.7E−03	3.7E−03	6.6E−05	4.2E−03	5.4E−02	6.9E−02
		SMT9	4.1E−03	5.0E−04	2.3E−03	4.9E−03	7.8E−05	5.0E−03	6.2E−02	7.9E−02
	Fishing port	SMS1	4.4E−03	1.2E−03	2.2E−03	4.8E−03	8.5E−05	5.2E−03	7.0E−02	8.8E−02
		SMS2	3.7E−03	7.4E−04	1.4E−03	2.6E−03	5.6E−05	3.4E−03	4.3E−02	5.4E−02
		SMS3	4.1E−03	3.8E−04	9.5E−04	2.9E−03	4.8E−05	3.0E−03	3.9E−02	5.1E−02
		SMS4	3.4E−03	1.2E−03	1.7E−03	2.2E−03	4.9E−05	2.6E−03	3.8E−02	4.9E−02
		SMS5	4.2E−03	1.3E−03	1.7E−03	2.1E−03	4.4E−05	2.5E−03	3.5E−02	4.6E−02
		SMS6	4.6E−03	8.6E−04	1.2E−03	1.3E−03	4.1E−05	1.6E−03	1.8E−02	2.8E−02
		SMS7	3.3E−03	1.2E−03	9.5E−04	2.6E−04	6.5E−06	1.3E−04	8.1E−04	6.7E−03
		SMS8	3.7E−03	1.7E−03	3.6E−04	2.9E−04	1.1E−05	1.6E−04	1.8E−04	6.4E−03
		SMS9	3.6E−03	4.7E−04	4.0E−03	2.4E−03	3.4E−05	6.5E−04	5.2E−03	1.6E−02

HI < 1, these findings suggest that health issues may not influence the area. This area’s sediments are entirely safe for human consumption. For ingestion, the value of HQ is ranked from highest to lowest as follows: Mn > Pb > Fe > Cd > Zn > Cu, while for dermal, it is Fe > Cu > Ni > Mn > Pb > Cd. We also see that the HI ingestion > HI dermal. Thus, these results indicate no health risk to these heavy metals when dermal is absorbed from the sediments [5, 20, 51].

The carcinogenic risk of Cd, Pb, and Ni per non-nutrition exposure in Red Sea sand was calculated, and the overall cancer risk over a lifetime (LCR) was indicated (Table 6). The LCR values for ingestion and dermal were (2.4 × 10^–6^ to 9.2 × 10^–5^) and (1.4 × 10^–5^ to 7.6 × 10^–5^) respectively. These findings suggest that the carcinogenic risk to human health from Red Sea sediments is not with acceptable limits (1 × 10^–6^ to 1 × 10^–4^) where the current values are very close to (4.7 × 10^–5^ to 1.6 × 10^–4^) at El-Qusier > (3.1 × 10^–5^ to 1.2 × 10^–4^) Safaga, respectively, which are close to the degrees of danger ([31], Nour et al., 2022a). The results show that the sediments from the research region have offer for cancer risks. This is disagreed with recent reports from the beaches of the Arabian Gulf, western Riyadh, the Gulf of Suez, and the Gulf of Aqaba (Nour et al., 2022a; [3, 4, 6], Al-Kahtany & El-Sorogy, 2023; [1]).

**Table 6:** Cancer Risks ingestion and dermal in area of investigation during 2021

Cancer risk ingestion							Cancer risk dermal				Total CSR
			Cd	Pb	Ni	CSR	Cd	Pb	Ni	CSR	
El Qusier	Fishing port	1	2.7E−07	9.4E−08	1.8E−05	1.9E−05	1.1E−06	2.3E−05	4.5E−06	2.9E−05	4.7E−05
		2	4.4E−07	6.5E−08	5.6E−05	5.6E−05	1.7E−06	1.6E−05	1.4E−05	3.2E−05	8.8E−05
		3	2.5E−07	9.4E−08	1.8E−05	1.8E−05	9.9E−07	2.3E−05	4.4E−06	2.9E−05	4.7E−05
		4	4.5E−07	9.1E−08	6.8E−05	6.8E−05	1.8E−06	2.3E−05	1.7E−05	4.1E−05	1.1E−04
		5	4.3E−07	8.4E−08	8.7E−05	8.8E−05	1.7E−06	2.1E−05	2.2E−05	4.4E−05	1.3E−04
		6	5.5E−07	1.1E−07	1.5E−05	1.6E−05	2.2E−06	2.8E−05	3.8E−06	3.4E−05	5.0E−05
		7	2.1E−07	9.8E−08	7.5E−05	7.6E−05	8.5E−07	2.4E−05	1.9E−05	4.4E−05	1.2E−04
		8	6.0E−07	1.3E−07	9.1E−05	9.2E−05	2.4E−06	3.1E−05	2.3E−05	5.6E−05	1.5E−04
		9	4.6E−07	9.0E−08	6.8E−05	6.9E−05	1.8E−06	2.2E−05	1.7E−05	4.1E−05	1.1E−04
		10	5.2E−07	7.4E−08	7.5E−05	7.6E−05	2.0E−06	1.8E−05	1.9E−05	3.9E−05	1.1E−04
		11	2.3E−07	1.0E−07	2.0E−05	2.0E−05	9.1E−07	2.6E−05	5.0E−06	3.2E−05	5.2E−05
	Desalination company	12	5.5E−07	2.1E−07	1.9E−05	2.0E−05	2.2E−06	5.3E−05	4.7E−06	6.0E−05	8.0E−05
		13	7.2E−07	1.9E−07	8.9E−05	9.0−05	2.9E−06	4.8E−05	2.2E−05	7.3E−05	1.6E−04
		14	8.2E−07	2.6E−07	1.1E−05	1.2E−05	3.3E−06	6.5E−05	2.7E−06	7.1E−05	8.3E−05
		15	6.8E−07	2.0E−07	1.5E−06	2.4E−06	2.7E−06	4.9E−05	3.7E−07	5.2E−05	5.5E−05
		16	4.1E−07	2.0E−07	3.6E−05	3.7E−05	1.6E−06	5.0E−05	9.0E−06	6.1E−05	9.7E−05
		17	4.5E−07	2.6E−07	4.2E−05	4.3E−05	1.8E−06	6.4E−05	1.1E−05	7.6E−05	1.2E−04
Safaga	Tourist port	1	1.4E−06	8.6E−08	7.9E−06	9.3E−06	5.4E−06	2.1E−05	1.9E−06	2.9E−05	1.4E−04
		2	1.8E−06	1.2E−07	8.4E−05	8.6E−05	7.0E−06	2.9E−05	2.1E−05	5.7E−05	8.2E−05
		3	1.1E−06	3.5E−08	5.4E−05	5.5E−05	4.3E−06	8.8E−06	1.3E−05	2.6E−05	5.9E−05
		4	1.3E−06	3.9E−08	3.4E−05	3.5E−05	5.2E−06	9.8E−06	8.4E−06	2.3E−05	7.3E−05
		5	9.9E−07	2.7E−08	4.9E−05	5.0E−05	3.9E−06	6.7E−06	1.2E−05	2.3E−05	8.1E−05
		6	1.4E−06	7.1E−08	4.5E−05	4.7E−05	5.5E−06	1.8E−05	1.1E−05	3.4E−05	6.3E−05
		7	1.3E−06	6.4E−08	3.3E−05	3.4E−05	5.1E−06	1.6E−05	8.2E−06	2.9E−05	5.8E−05
		8	1.4E−06	6.2E−08	2.9E−05	3.0E−05	5.4E−06	1.5E−05	7.1E−06	2.8E−05	6.0E−05
		9	1.3E−06	2.6E−08	3.8E−05	3.9E−05	5.3E−06	6.4E−06	9.4E−06	2.1E−05	6.7E−05
	Fishing port	10	1.4E−06	6.2E−08	3.6E−05	3.7E−05	5.7E−06	1.5E−05	8.8E−06	3.0E−05	4.4E−05
		11	1.2E−06	3.8E−08	2.3E−05	2.4E−05	4.8E−06	9.4E−06	5.7E−06	2.0E−05	3.1E−05
		12	1.4E−06	1.9E−08	1.6E−05	1.7E−05	5.4E−06	4.8E−06	3.9E−06	1.4E−05	5.5E−05
		13	1.1E−06	6.2E−08	2.7E−05	2.8E−05	4.4E−06	1.5E−05	6.7E−06	2.6E−05	5.8E−05
		14	1.4E−06	6.5E−08	2.8E−05	3.0E−05	5.5E−06	1.6E−05	7.0E−06	2.9E−05	4.3E−05
		15	1.5E−06	4.4E−08	2.0E−05	2.1E−05	6.0E−06	1.1E−05	4.9E−06	2.2E−05	4.0E−05
		16	1.1E−06	6.2E−08	1.6E−05	1.7E−05	4.3E−06	1.5E−05	3.9E−06	2.4E−05	3.5E−05
		17	1.2E−06	8.7E−08	6.0E−06	7.3E−06	4.9E−06	2.2E−05	1.5E−06	2.8E−05	9.3E−05
		18	1.2E−06	2.4E−08	6.5E−05	6.6E−05	4.7E−06	6.1E−06	1.6E−05	2.7E−05	9.3E−05

### Data analysis

#### Cluster analysis (CA)

**Fig. 5:**
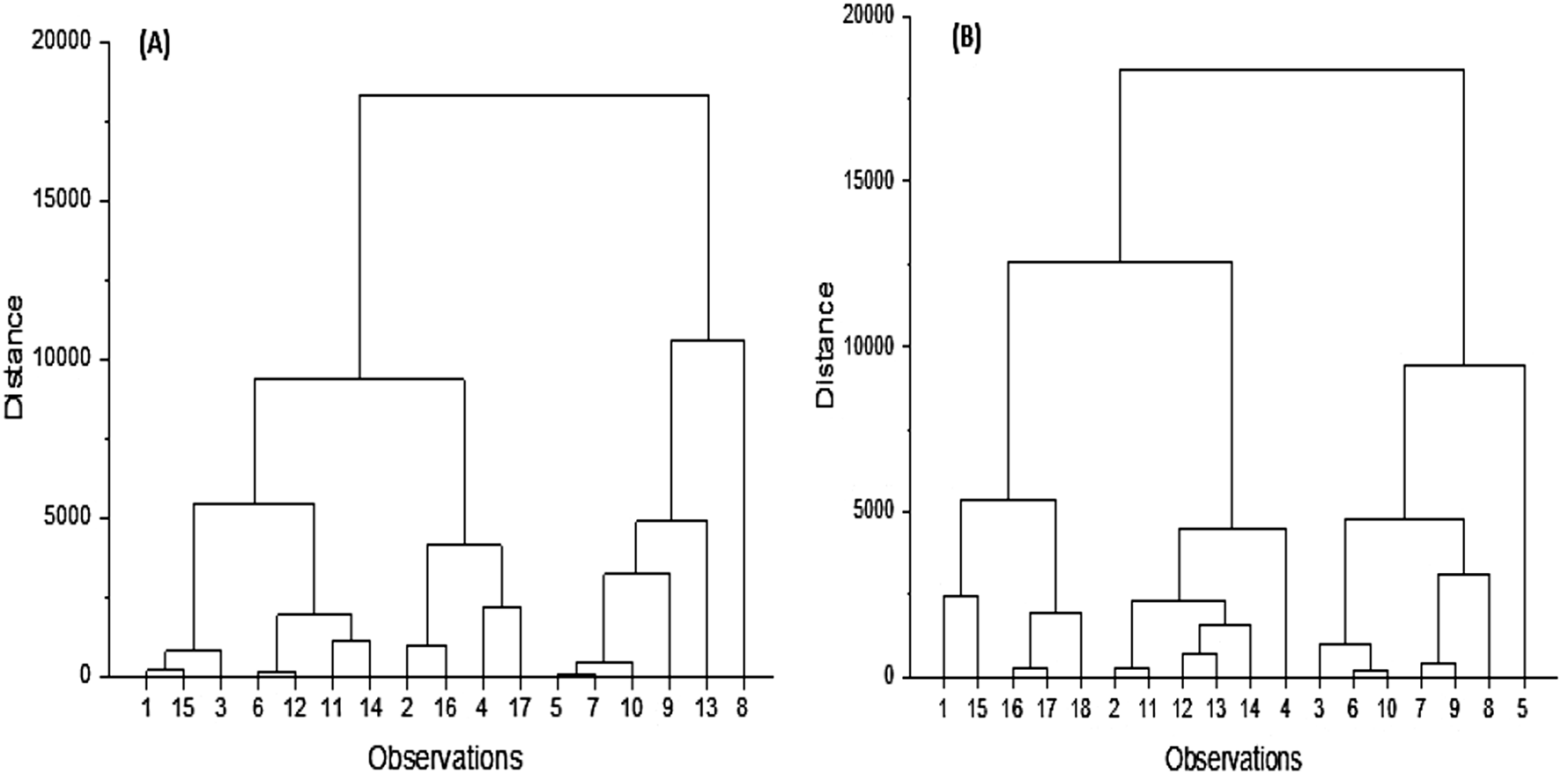
Hierarchical cluster analysis in El-Quseir (**A**) and Safaga sector (**B**)

The clustered tree diagram demonstrates that most sampling sites have comparable heavy metal distributions. The similarity analysis of El-Quseir locations (Fig. 5) reveals relative relationships among land uses and activities based on metal ion analysis. According to the results of CA in El-Quseir sections, eight statistically significant clusters were formed. Sampling sites 8 corresponded to 9, 10, 13, 5, and 7 stations. While in the Safaga area, the cluster (station 5) corresponded to stations 8, 9, 7, 10, 6, and 3, which have similar properties. However, these areas are disposed to fishing, tourism, and phosphate loading operations. El-Quseir localities are linked in the same cluster because of the same sources of oil pollution and desalination processes [34]. Fishing operations, sewage effluents, and tourism activities all impact Safaga places. The sampling sites of 1 and 3, 11 and 12, 16 and 17, 7 and 9, and 10 and 13 were identical. Saleem et al., 2018 discovered a high concentration of HMs at sites near urban and semi-urban areas. Cd, Cr, Cu, Ni, and Pb are all derived primarily from natural sources. The influence of areas immediately close to reservoirs is also significant,additionally, in the case of Ni, the number of road and river crossings has a vital role [49].

#### Correlation analysis

The interrelationships matrices between the studied elements in the coastal sediments between Safaga and El-Quseir—Red Sea, Egypt, are calculated and are shown in Table 7. Various degrees of correlations were found. There are some significant correlations, both positive and negative, between the heavy metals in the study area. In the El-Quseir sector, Zn shows a good relationship with Cu and Ni (r = 0.864 and 0.959) and negative with Cd and Pb. Mn shows a better relationship with Ni, Cu, K (r = 0.932, r = 0.909, r = 0.975). He shows a positive relationship with Ni, Cu, Zn, and Mn (r = 0.950, r = 0.961, r = 0.945 and 0.955) while showing an insignificant relationship with other elements. However, in the Safaga sector, Mn and Zn show a positive relationship with Cu (r = 0.873 and 0.971), respectively. At the same time, the Zn shows a positive significant correlation to Cu (r = 0.971). Zinc is frequently found with other metals such as copper, lead, and cadmium.

**Table 7:** Correlation coefficients between the heavy metals in of coastal sediments from the Safaga and El-Quseir-Red sea, Egypt

El-Quseir city								
	Cd	Pb	Ni	Co	Cu	Zn	Mn	Fe
Cd	1							
Pb	0.577*	1						
Ni	0.003	− 0.369	1					
Co	0.248	0.163	0.301	1				
Cu	0.155	− 0.121	0.865**	0.579*	1			
Zn	0.044	− 0.245	0.959**	0.288	0.864**	1		
Mn	0.065	− 0.201	0.932**	0.416	0.909**	0.975**	1	
Fe	0.155	− 0.194	0.950**	0.492*	0.961**	0.945**	0.955**	1

Safaga city								
	Cd	Pb	Ni	Co	Cu	Zn	Mn	Fe
Cd	1							
Pb	0.522*	1						
Ni	0.216	0.05	1					
Co	− 0.239	− 0.076	0.490*	1				
Cu	− 0.19	− 0.34	0.490*	0.673**	1			
Zn	− 0.128	− 0.33	0.412	0.557*	0.971**	1		
Mn	0.09	− 0.179	0.358	0.39	0.873**	0.913**	1	
Fe	− 0.046	− 0.21	0.409	0.584*	0.949**	0.966**	0.957**	1

^*^Correlation is significant at the 0.05 level (2-tailed)

^**^Correlation is significant at the 0.01 level (2-tailed)

## Conclusion

Metal contamination indices help identify metal toxicity effects at monitored sites. The findings, which include pollution indices and correlations demonstrating how metals affect the stations under study, show that most heavy metals and pollutants in Red Sea sediments come from natural sources, with only a small amount coming from human activities such as oil and phosphate mining. Numerous environmental contamination indicators in this study suggest that Cd poses a significant ecological concern to the Red Sea shoreline in the examined region, particularly in the Quseir area. Cd and Pb levels are high in the Safaga region. This study provides updated data on heavy metal contamination levels in Red Sea marine sediments, making its findings relevant and valuable for future research and economic growth. Furthermore, the current findings offer a helpful foundation for identifying regional standards. Heavy metal contamination concentrations along the Red Sea coast must be monitored to reduce ecological concerns.

### Supplementary Information

**Additional file1: Table S1** Exposure parameters used for the health risk assessment through different exposure for pathways for soil USEPA, [53].

## Data Availability

The raw data supporting the conclusions of this manuscript would be available by the authors, without undue reservation, to any qualified researcher.
