# Supplementary material for: A comparative study of the risk assessment and heavy metal contamination of coastal sediments in the Red sea, Egypt, between the cities of El-Quseir and Safaga
Source: Geochem Trans. 2024 May 3;25:3. doi: 10.1186/s12932-024-00086-8 (PMC11069267; doi:10.1186/s12932-024-00086-8)
Supplement: Supplementary file 1 — Additional file1: Table S1 Exposure parameters used for the health risk assessment through different exposure for pathways for soil USEPA, [53]. [file 12932_2024_86_MOESM1_ESM.docx]

**Appendix**

Table (S1): Exposure parameters used for the health risk assessment through different exposure for pathways for soil (USEPA, 2012).

| **Parameters** | **Unit** | **Adults** |
| --- | --- | --- |
| Ingestion Rate IRs | mg/day | 100 |
| Conversion Factor CF | Kg/mg | 10^−6^ |
| Exposure Duration ED | Years | 30 |
| Exposure Frequency EF | Day/year | 350 |
| Body Weight BW | Kg | 70 |
| Dermal Absorption from Sediment ABS |  | 0.1 |
| Exposed Skin Surface Area SA | Cm^2^ | 5800 |
| Skin Adherence Factor SL | mg cm^−2^h^−1^ | 0.61 |
| Adherence Factor from sediment to skin AF | mg/cm^2^ | 0.07 |
| Cancer Slop Factor (Ingestion) | mg/kg/day | Cd= 0.38, Pb= 8.5x 10^-3^, Ni = 1.7 |
| Cancer Slop Factor (Dermal) |  | Cd= 6.1, Pb= 8.5 |
| Reference Dose (RfD) Ingestion |  | Cd=5x 10^-4^, Pb= 4x10^-3^, Ni= 2x 10^-2^  Cu= 4x 10^-2^, Zn= 0.06, Fe= 7 x 10^-1^ |
| Reference Dose (RfD) Dermal |  | Cd=0.005, Pb= 3.5x 10^-3^, Ni= 5.6 x 10 ^-3^, Cu 2.4x 10^-2^, Mn= 0.024, Zn=3 x 10^-1^ , Fe= 0.14 |
| Average Time AT:  For non-carcinogens,  carcinogens | Days | ED × 365  70 x 365=25550 |
